# G Protein-Coupled Estrogen Receptor 1 Mediates Acute Estrogen-Induced Cardioprotection via MEK/ERK/GSK-3β Pathway after Ischemia/Reperfusion

**DOI:** 10.1371/journal.pone.0135988

**Published:** 2015-09-10

**Authors:** Mohammad E. Kabir, Harpreet Singh, Rong Lu, Bjorn Olde, L. M. Fredrik Leeb-Lundberg, Jean Chrisostome Bopassa

**Affiliations:** 1 Department of Physiology, School of Medicine, University of Texas Health Science Center at San Antonio, TX 78229, United States of America; 2 Department of Pharmacology and Physiology, Drexel University College of Medicine, Philadelphia, PA 19102, United States of America; 3 Department of Anesthesiology, Division of Molecular Medicine, University of California Los Angeles, Los Angeles, CA 90095–1778, United States of America; 4 Department of Experimental Medical Science, Lund University, 22184 Lund, Sweden; Indiana University School of Medicine, UNITED STATES

## Abstract

Three types of estrogen receptors (ER) exist in the heart, Esr1, Esr2 and the G protein-coupled estrogen receptor 1, Gper1. However, their relative importance in mediating estrogen protective action is unknown. We found that, in the male mouse ventricle, Gper1 transcripts are three- and seventeen-fold more abundant than Esr1 and Esr2 mRNAs, respectively. Analysis of the three ER knockouts (Esr1^-/-^, Esr2^-/-^ and Gper1^-/-^) showed that only the Gper1^-/-^ hearts lost their ability to be protected by 40 nM estrogen as measured by heart function, infarct size and mitochondrial Ca^2+^ overload, an index of mitochondrial permeability transition pore (mPTP) activity. Analysis of Akt, ERK_1/2_ and GSK-3β salvage kinases uncovered Akt and ERK_1/2_ transient activation by estrogen whose phosphorylation increased during the first 5 min of non-ischemic perfusion. All these increase in phosphorylation effects were abrogated in Gper1^-/-^. Inhibition of MEK_1/2_/ERK_1/2_ (1 μM U0126) and PI-3K/Akt (10 μM LY294002) signaling showed that the MEK_1/2_/ERK_1/2_ pathway *via* GSK-3β exclusively was responsible for cardioprotection as an addition of U0126 prevented estrogen-induced GSK-3β increased phosphorylation, resistance to mitochondrial Ca^2+^-overload, functional recovery and protection against infarction. Further, inhibiting PKC translocation (1 μM chelerythrin-chloride) abolished estrogen-induced cardioprotection. These data indicate that estrogen-Gper1 acute coupling plays a key role in cardioprotection against ischemia/reperfusion injury in male mouse *via* a cascade involving PKC translocation, ERK_1/2_/GSK-3β phosphorylation leading to the inhibition of the mPTP opening.

## Introduction

Estrogen (17β-estradiol, E2) is well known for its protective action on cardiovascular function. E2 effects can be mediated by three types of estrogen receptors (ERs), ER alpha (Esr1 or ERα), ER beta (Esr2 or ERβ) and the G-protein coupled estrogen receptor 1 (Gper1, referred to as GPR30). All of these receptors have been detected in the heart [1–3], where acute application of E2 prevents damage from ischemia/reperfusion (I/R) injury [4,5].

Efforts to discern the role of each of the ERs in protecting the heart from I/R injury after acute E2 treatment have been mostly pharmacologically based and support Esr1 and Gper1 as mediators of the rapid action of E2 but the role of Esr2 is unclear. Specifically in male rats, Esr1 and Esr2 participation is supported by the mimetic actions of their respective agonists 4,4’,4”-[4-Propyl-(1H)-pyrazole-1,3,5-triyl]tris-phenol, PPT, and, 2,3-bis(4-hydroxyphenyl)-propionitrile, DPN, in protecting the heart from ischemia/reperfusion [6]; while studies in hearts of female rabbits using the same drugs discarded the role of Esr2 but agreed on the role of Esr1 [7]. In addition, acute E2 treatment has been found to induce similar reno-protective effect in WT, Esr1^-/-^ and Esr2^-/-^ after cardiac arrest and cardiopulmonary resuscitation, suggesting an independent of these two ERs mechanism [8]. On the other hand, we and others have found that Gper1 agonist, G1 is also able to protect the heart against I/R injury in male mice and in rats of either gender [3,9–11]. However, recent studies have shown that G1 may have alternative effects independent of Gper1 activation [12]. Thus, the questions remain on the role of Gper1 and on which of the receptors is of major importance in the acute action of E2 in the heart.

Gper1 signaling pathways are beginning to emerge, with the majority of the studies performed in cancer cell lines where cAMP and ERK_1/2_ activation play a role [13]. In the heart, only a couple of studies (including ours) have addressed the signaling pathways triggered by stimulation of Gper1 by G1 [3,9]. However, the two studies used different protocols in the timing of G1-stimulation leading to different conclusions about the role of ERK_1/2_ pathway in the acute protective action that G1 (or E2) has on the heart from ischemia/reperfusion injury.

E2 short-term action should be favored by localization of ERs at the plasma membrane. In fact, in heterologous expression systems the rapid action of E2 is enabled by the activation of Esr1 and Esr2 tethered to the plasma membrane *via* palmitoylation with subsequent triggering of kinase signaling cascades [14]. In the heart, Esr2 signals are significant in nuclear, cytosolic and mitochondrial fractions but absent in the sarcolemma [15,16]. In contrast, Esr1 besides being in the nucleus and cytosol it is also localized to the plasmalemma and T-tubules of cardiomyocytes [17], raising the probability of this receptor playing a role in the acute E2-mediated protection against I/R injury.

Because G protein-coupled receptors are classically localized to the plasma membrane it is reasonable to hypothesize that Gper1 and/or Esr1 are the main targets for the acute effect of E2 in the heart preventing I/R injury. Here, we quantified ERs transcript expression, and investigated the role of each of the receptors by using Gper1, Esr1 and Esr2 knockout animals. We found that Gper1 expression is critical for acute estrogen action and thus, further investigated the signaling mechanisms involved in this E2-triggered cardioprotective pathway.

## Materials and Methods

### Animals

Male 2–3 mo old mice were used: wild type (C57BL/6NCrl), Esr1^-/-^, Esr2^-/-^ [18]and Gper1^-/-^ [2]. Protocols received UT Health Science Center at San Antonio Institutional Animal Care and Use Committee (IACUC) institutional approval.

### Langendorff preparation and I/R protocol

Mice were injected intraperitoneally with the anesthetic pentobarbital (60 mg/kg) together with heparin (200 UI/kg) to prevent blood coagulation. Once the animals were completely anesthetized, the hearts were removed after a trans-abdominal incision and the diaphragm cut to expose the thoracic cavity. Hearts were immediately immersed and arrested in cold (4°C) Krebs-Henseleit bicarbonate buffer (KH) (mM): 11.1 glucose, 118 NaCl, 4.7 KCl, 1.2 MgSO_4_, 1.2 KH_2_PO_4_, 25 NaHCO_3_, 2 CaCl_2_, pH 7.4. The aorta was rapidly cannulated and the heart retrograde-perfused at a constant rate (3 ml/min) in the Langendorff apparatus using KH buffer with drugs or with vehicle bubbled with 95% O_2_ + 5% CO_2_ at 37°C. A well-established ex-vivo I/R injury protocol was used [3,19–21] consisting of the following: Hearts were stabilized suspended in the humid chamber at 37°C for 20–40 min to reach quasi steady-state contractility (left ventricular developed pressure, LVDP ~ 80 mmHg). After stabilization, hearts were subjected to 18 minutes of global normothermic ischemia by clamping the aorta and immersing the hearts in KH at 37°C in the water-jacketed chamber, and reperfused suspended in the humid chamber at 37°C for 10 min for biochemical characterization, or for 60 min to monitor functional recovery and determine infarct size at the conclusion of the experiment. We preferentially considered good hearts the one that reaches a minimum left ventricular developed pressure of 80 mmHg at the end of the basal perfusion (Before ischemia). In the mouse model, this I/R protocol typically results in ~50% of infarct size. Sham hearts were not subjected to I/R but only perfused for ~120 min (duration of the I/R protocol). Estrogen alone or with addition of different inhibitors mentioned below was given from the beginning and throughout the experiment.

### Functional measurements

The heart function was recorded throughout the experiment using a catheter (1.4F SPR-671; Millar Instruments, Colorado Springs, CO) connected to a MAC LAB (Powerlab) acquisition system from ADInstruments. The catheter was directly inserted into the left ventricle (LV) after a left atrial incision was made to expose the mitral annulus for recording the LV end-systolic pressure (LVSP), the LV end-diastolic pressure (LVEDP), and heart rate as described in our article (3). Note that the maximum rate of rise of the LV pressure (dP/d*t*_max_) and the maximum isovolumetric rate of relaxation (dP/dt_min_) values were directly obtained from LabChart5.5 (ADInstruments) software. The LV developed pressure (LVDP = LVESP - LVEDP) and Rate Pressure Product (RPP = LVDP x HR) were calculated from the recordings every ten minutes.

### Myocardial infarct size

The heart was removed from the Langendorff apparatus at the end of the reperfusion period, and cut into four transverse slices parallel to the atrio-ventricular groove as previously described in [3]. After removing the right ventricular tissue, slices were incubated for 10 min in 1% triphenyltetrazolium chloride (TTC) at 37°C followed by fixation with 4% paraformaldehyde. This staining differentiates the infarcted (pale) from viable (red) myocardial tissue. The slices were photographed using digital microscopic imaging. The area of necrosis was quantified by computerized planimetry with Adobe Photoshop. Total area of necrosis was calculated and expressed as the percentage of total left ventricular area.

### Ca^2+^-induced opening of the mitochondrial permeability transition pore (mPTP)

#### Mitochondria isolation

At 10 min of reperfusion, the hearts were arrested in cold KH and the right ventricle discarded. All procedures were carried out at 4°C. The left ventricle (approximately 0.15–0.22 g) was placed in isolation buffer A (mM): 70 sucrose, 210 mannitol, 1 EDTA and 50 Tris-HCl, pH 7.4. The tissue was finely minced with scissors and homogenized in the same buffer A (1 ml buffer/0.1 g of tissue) using Kontes and Potter-Elvehjem tissue grinders. The homogenate was centrifuged at 1,300 xg for 3 min, and the supernatant was filtered through cheesecloth and centrifuged at 10,000 xg for 10 min. The supernatant was discarded and the pellet was gently washed 3 times with 500 μl of buffer B (in mM): 150 sucrose, 50 KCl, 2 KH_2_PO_4_, 5 succinic acid, and 20 Tris/HCl, pH 7.4, and resuspended with 50 μl of the same buffer. Mitochondrial protein concentration was assayed using the Bradford method and adjusted to a final concentration of 25 mg/ml.

#### Calcium Retention Capacity (CRC)

Mitochondrial CRC was measured spectroscopically using calcium green-5N (Invitrogen) and excitation and emission wavelengths set at 500 and 530 nm, respectively. Changes in fluorescence were expressed as ΔF/F and were calculated by dividing each fluorescence point by the maximum fluorescence (average of 30 s just before mitochondria loading). Isolated mitochondria (500 μg of mitochondrial protein) were added to a spectrofluorometer cuvette containing 2 ml of buffer B supplemented with 0.5 μM calcium green-5N under constant stirring. Upon addition of mitochondria, there is a progressive reduction of the Ca^2+^ in the media due to mitochondrial Ca^2+^ uptake reaching a quasi-steady-state in ~90 s. At this time, Ca^2+^ pulses of 10 nm (2 μl of 5 mM CaCl_2_ stock solution) were added every 60 s to the 2 ml spectrophotometer cuvette. The Ca^2+^ pulses induced a peak of extra-mitochondrial Ca^2+^ concentration that returns to near-baseline levels as Ca^2+^ enters the mitochondrial matrix. With increasing mitochondria calcium loading, extra-mitochondrial Ca^2+^ starts accumulating until the addition of Ca^2+^ leads to a sustained Ca^2+^ increase indicating a massive release of mitochondrial Ca^2+^. CRC was defined as the amount of Ca^2+^ (normalized to mitochondrial protein) load required to induce the massive Ca^2+^ release, which corresponds to mPTP opening. To validate this point, we have performed CRC measurements in absence (control) or presence of Cyclosporin A (CsA), an inhibitor of mPTP, and using mitochondria from freshly isolated hearts. In the presence of 2 μM CsA, mitochondria can endure more Ca^2+^ load than the control mitochondria (CRC_control_ = 273±18 nmol Ca^2+^/mg protein, n = 3; CRC_CsA_ = 380 ± 20 nmol Ca^2+^/mg protein, n = 3).

### RNA extraction, cDNA synthesis and real-time PCR amplification

Total RNA was extracted from mouse heart ventricles with Trizol reagent (Invitrogen) followed by DNase digestion for 10 min at room temperature with RNase-Free DNAse Set (Qiagen), and cleaned-up with RNeasy Mini Kit (Qiagen). The quality of the RNA samples was determined by electrophoresis through agarose gels; only RNA samples with 28S:18S rRNA ratio ≥2 were used.

Oligo-dT primer was used to target mRNAs present in the total RNA samples for conversion into cDNAs by reverse transcriptase (RT). Cleaned-up total RNA (2 μg) was reverse transcribed in a final volume of 20 μl containing 1xRT buffer, 0.5 mM dNTP Mix, 10 units RNasin RNase inhibitor (Promega), 4 units Omniscript RT (Qiagen) and 1 μM oligo-dT primer. Samples were incubated at 37°C for 60 min followed by RT inactivation at 95°C for 5 min. As negative control, mock cDNA was prepared in parallel in the same reaction solution but lacking Omniscript RT (RT negative,-RT). The quality of cDNA was confirmed by the lack of detectable genomic DNA after PCR using primers flanking two introns (1046 bp) of mouse β-actin (NM_007393.3) (forward primer, 5′-TCCTTCGTTGCCGGTCCACA-3′, nucleotides 43–62, reverse primer, 5′-CCTCTCTTGCTCTGGGCCTCG-3′, nucleotides 247–267); the expected cDNA product size is 225 bp.

Real-time PCR and gene specific primers were used for quantification of Esr1, Esr2 and Gper1cDNAs using iQ^TM^ SYBR Green Supermix (Bio-Rad). Esr1 and Esr2 primers spanned an intron. Primers were: for Esr1 (NM_007956.4), forward primer 5′-CGAAGTGGGCATGATGAAAGG-3′, (nucleotides 931–951) and reverse primer 5′-AAGGACAAGGCAGGGCTATTC-3′, (nucleotides 1102–1122), expected product of 192 bps; for Esr2 (NM_010157.3), forward primer 5′-GCCAACCTCCTGATGCTTCTT-3′ (nucleotides 1770–1790) and reverse primer 5′-TTGTACCCTCGAAGCGTGTGA-3′ (nucleotides 1895–1915), expected product of 146 bps; and for Gper1 (NM_029771.3), forward primer 5′-CTGCAAGCAGTCTTTCCGTCA-3′ (nucleotides 1489–1509) and reverse primer 5′-GCTCGTCTTCTGCTCCACATA-3′ (nucleotides 1622–1642), expected product of 154 bps. All primers were designed with primer-BLAST (NCBI) and OligoAnalyzer 3.1 (Integrated DNA Technologies, IDT). The specificity of the primers was checked by the BLAST program. The thermal cycling conditions comprised an initial denaturation step at 95°C for 3 min, and 40 cycles at 95°C for 45 s, 61°C for 45 s, and 72°C for 45 s. Negative controls included parallel reactions with mock cDNA or with H_2_O instead of template. Positive controls included amplification of Esr1 and Esr2 subcloned into pcDNA3, and Gper1 subcloned into p3XFLAG-CMV-14 (called here plasmid DNA). Another quality control was the relative abundance of the housekeeping gene, β-actin with typical threshold cycle of ~15. Specific products were detected as clear single peaks at their melting temperature in the first derivative of fluorescence (dF/dT) *versus* temperature plot (melting curve). As expected from the melting curve, a single band of the expected size was detected in agarose gel electrophoresis at the end of the reaction. Esr1, Esr2 and Gper1 standard curves were obtained using known concentrations of linearized Esr1, Esr2 and Gper1 plasmid DNAs; a threshold was assigned in the linear range of the fluorescence vs. cycle number plot and the threshold cycle was plotted as a function of linearized plasmid DNA. The data were fitted to a straight line, where its slope is an indication of the reaction efficiency: % efficiency = (10^−1/slope^− 1)*100. Experimental points were interpolated to obtain absolute cDNA values and data was expressed as pg cDNA/μg total RNA.

### Immunoblot analysis

Whole hearts were snap-frozen in liquid N2, then powdered, and resuspended in the lysis buffer containing (mM): 150 NaCl, 50 Tris-HCl, 0.1% NP-40 alternative, 5 EDTA-Na_2_, 10 HEPES, 0.25% Na-deoxycholate, 1 Na_3_VO_4_, 0.5 NaF, pH 7.4 supplemented with Protease Inhibitor Cocktail (Roche, 1 tablet/10 ml). The lysates were centrifuged at 12,000 xg for 5 min and the supernatants were collected. For electrophoresis, 30 μg of protein/lane was loaded on a 10% Tris/HCl SDS polyacrylamide gel. Proteins were electrotransferred to a nitrocellulose membrane and then blocked with 5% non-fat dry milk in 20 mM of Tris buffered saline with 0.1% Tween-20 for 60 min. Membranes were then incubated overnight at 4°C with primary antibodies against: pAkt (Ser473), 33 ng/ml; pGSK-3β (Ser9), 67 ng/ml; pERK_1/2_ (phospho-p44/42 MAPK-ERK_1/2_-Thr202/Tyr204), 0.31 μg/ml; GSK-3β, 24 ng/ml; Akt, 38 ng/ml, or p44/42 MAPK (ERK_1/2_), 19 ng/ml and vinculin, 20 ng/ml (from Santa Cruz) was used as a loading housekeeping protein. After washing, membranes were incubated for 1 h at room temperature with the corresponding fluorophore-conjugated secondary antibodies (goat anti-rabbit Alexa 680, 20 ng/ml; goat anti-mouse IR Dye 800CW, 10 ng/ml). After washing, bands were visualized using an infrared fluorescence system (Odyssey Imaging System, Li-COR Biosciences).

### Pharmacological Agents and Antibodies

17β-estradiol and chelerythrine chloride (CC) were from Sigma-Aldrich (St. Louis, MO). 2-(4-Morpholinyl)-8-phenyl-4H-1-benzopyran-4-one (LY 294002) and U0126 were from Invitrogen. All primary antibodies were from Cell Signaling. Except for E2, which was diluted in ethanol (final ethanol concentration in perfusion buffer was <0.001%), all of the other pharmacological agents were dissolved in DMSO (final DMSO concentration in perfusion buffer was 0.01%). We previously showed that this concentration of DMSO does not affect heart function or myocardial infarct size after I/R [22]. We also did not present in this manuscript the control inhibitors groups because we already showed that at the concentrations used, all these inhibitors did not change the heart functional recovery and myocardial infarction as compared to normal controls [3,22]

### Statistical Analysis

Error bars are the standard errors of the mean (±SEM) for a minimum of three independent hearts (n≥3). For cardiac infarct size and mitochondrial CRC means were compared between groups using one-way ANOVA. For the *ex vivo* functional studies, mean profiles over time were compared across groups using repeated-measure ANOVA methods. Under the ANOVA model, pair wise mean comparisons were judged significant using the Turkey studentized range criterion. SPSS, version 13.0 (SPSS Inc, Chicago, IL) was used to carry out the computations. Because all outcomes were continuous, results were summarized with means ± SEMs. *P* < 0.05 was considered statistically significant.

## Results

### Gper1 mRNAs are the most abundant ER transcripts in male mouse ventricle

**Fig 1:**
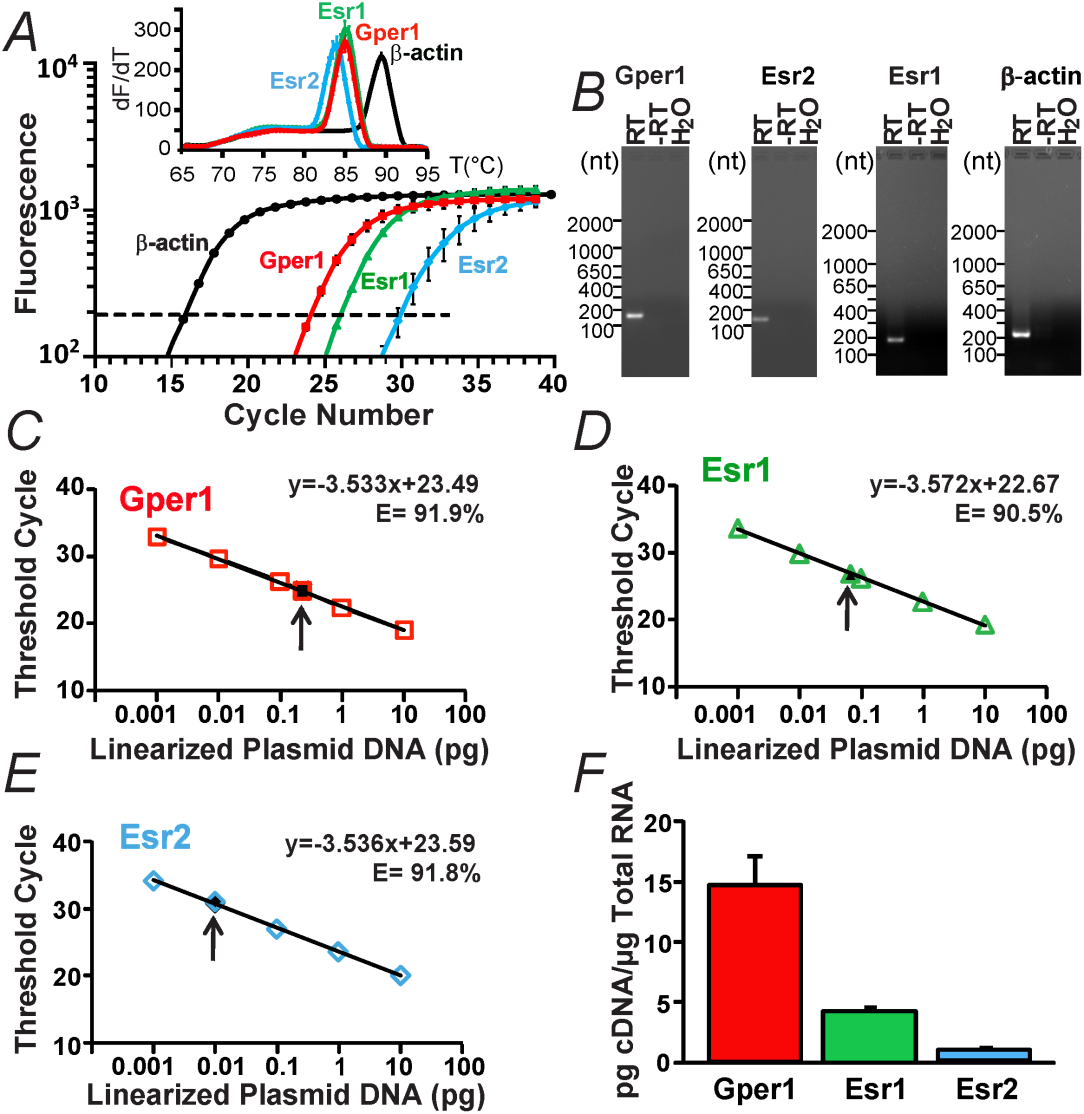
Gper1 transcripts predominate over Esr1 and Esr2 in male mouse heart. **A.** An example of relative fluorescence intensity versus PCR cycle number for Esr1, Esr2, Gper1 and β-actin (housekeeping gene) in mouse ventricle. Inset, melting curves for Gper1, Esr1, Esr2, and β-actin reactions. **B.** Real-time PCR end-products for Gper1, Esr1, Esr2 and β-actin with reverse transcriptase (RT), without RT (-RT) and water instead of total RNA (H_2_O). **C-E.** Standard calibration curves for Gper1, Esr1, and Esr2 DNA plasmids, respectively. Open symbols, known amount of linearized plasmid DNA. Closed symbols, interpolated absolute values of estrogen receptor cDNAs in mouse heart (arrow). **F.** Mean absolute values of Gper1, Esr1 and Esr2 transcript levels in mouse heart. Quantifications were performed in duplicate from samples of three different animals (n = 3). In this and following figures, data were acquired from male mice.

The abundance of ERs transcripts in heart ventricles was investigated using quantitative real-time PCR (qPCR). Fig 1A shows that Gper1, Esr1, and Esr2 are differentially expressed in ventricles with Gper1 signals appearing earlier at lower PCR cycles than Esr1 or Esr2. The fluorescence vs. cycle number plot also displays the amplification of the house-keeping gene, β-actin (control). The corresponding melting curves (insert) show a single peak for each product indicating that single specific products were amplified. This was further confirmed by analysis of the qPCR end-products using agarose gel electrophoresis, which showed a single band of the expected molecular sizes (Fig 1B, RT). Fig 1B also shows the absence of products in negative controls using mock cDNA (-RT) or H_2_O instead of cDNA. Fig 1C–1E shows the standard curves for Gper1, Esr1, and Esr2 using plasmid DNA (open symbols) together with the experimental values of heart samples marked with an arrow (filled symbols). The plasmid data (open symbols) were well fitted to a straight line (R^2^ = 0.999) with slopes around 3.5 demonstrating that the estrogen receptor primers used in this study have similar efficiencies (E) of 91.9% (Gper1), 90.5% (Esr1), and 91.8% (Esr2). Absolute cDNA values of each estrogen receptor were interpolated from the corresponding standard curves and expressed per μg of total RNA used. Values were: Gper1 = 14.55±2.54 pg cDNA/μg total RNA (n = 3), Esr1 = 4.27±0.29 pg cDNA/ μg total RNA (n = 3), and Esr2 = 0.87±0.38 pg cDNA/μg total RNA (n = 3) (Fig 1F). Assuming a 100% conversion of mRNA to cDNA, our present data indicate that Gper1 transcripts are 3 fold more abundant than Esr1 and near 17 fold more numerous than Esr2 in male mouse ventricle.

### Gper1-but not Esr1 nor Esr2- activation is essential for acute E2-mediated heart protection from ischemic/reperfusion (I/R) injury

To evaluate the role of Esr1, Esr2 and Gper1 receptors in acute E2-induced cardioprotection, hearts from WT and KO mice were subjected to an I/R protocol in the continuous presence of 40 nM E2 or vehicle (control) in the perfusate. For comparison, we also analyzed the activity of the isolated heart without interventions (sham).

**Fig 2:**
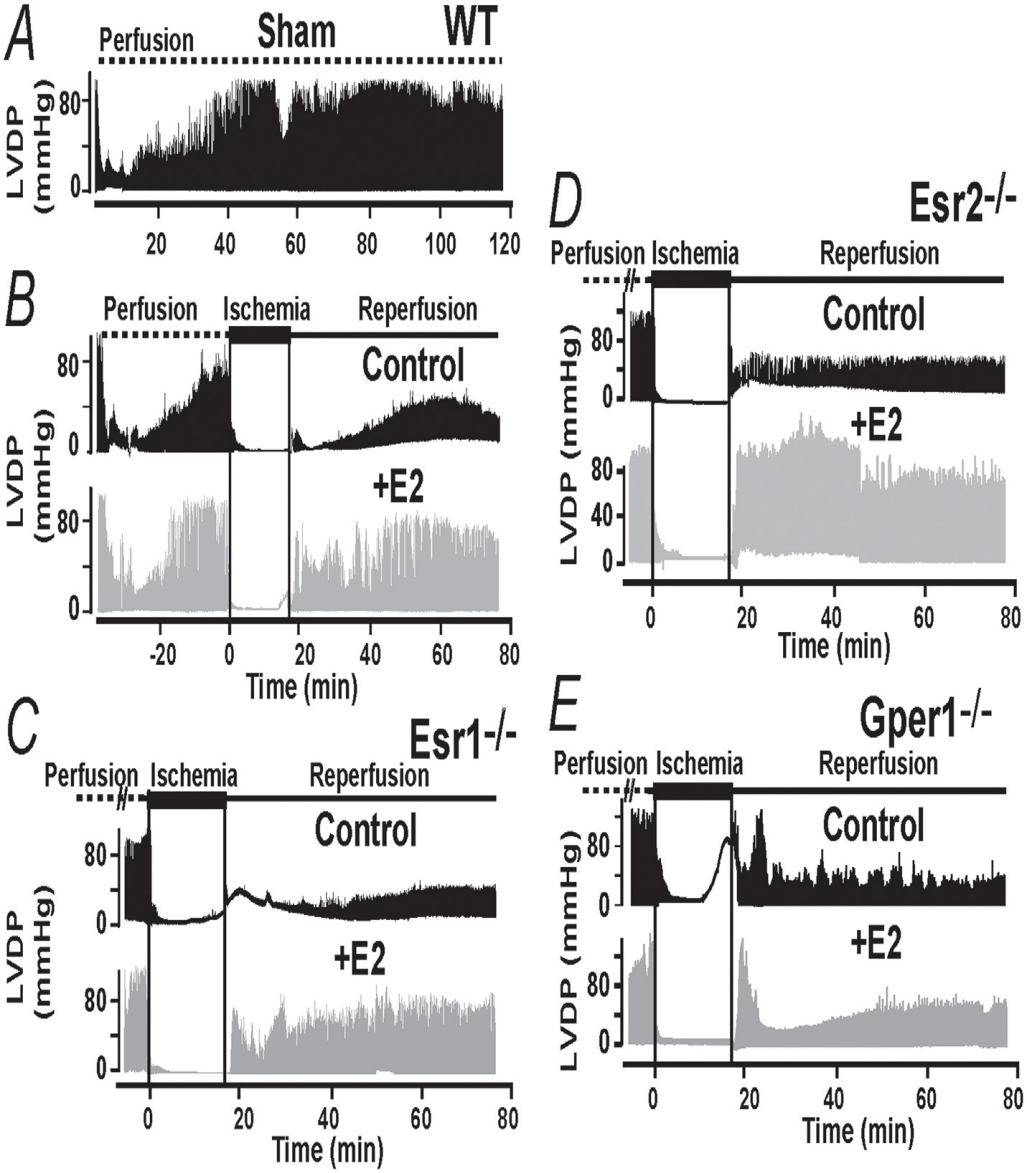
Gper1 activation is essential for E2-mediated cardioprotection. **A.** Recordings of left ventricular developed pressure (LVDP) comparing heart activity from wild-type (WT) animals not subjected to ischemia/reperfusion (Sham) with the function of hearts subjected to the ischemia reperfusion (I/R) protocol under the continuous presence of vehicle (control) or 40 nM E2 in KH buffer. In sham hearts, function stabilizes at about 30–40 min after mounting and is stable for at least 2 hours. In hearts subjected to I/R (middle and bottom traces), E2 improved recovery from ischemia (during reperfusion) compared to control. **B-D.** LVDP traces from hearts of Esr1, Esr2 and Gper1 knockouts (-/-) subjected to I/R. Only in Gper1^-/-^ hearts, E2 lost its ability to improve functional recovery (**E**). n = 6–8 hearts/ group.

The top traces in Fig 2A correspond to an isolated WT heart without interventions and show that after a period of stabilization, in this case 40 min, the heart activity measured as left ventricular developed pressure, LVDP, is maintained stable for at least another 80 min. In contrast and as reported earlier [3,10,23], if a WT male heart is subjected to a period of ischemia followed by reperfusion (Fig 2B, black trace), during reperfusion the heart function recovers but only to a certain degree typically less than 40% (at ~60 min from reperfusion onset). This heart functional recovery is dramatically improved by E2, which usually reaches 75% of its original activity (Fig 2B, gray traces).

**Fig 3:**
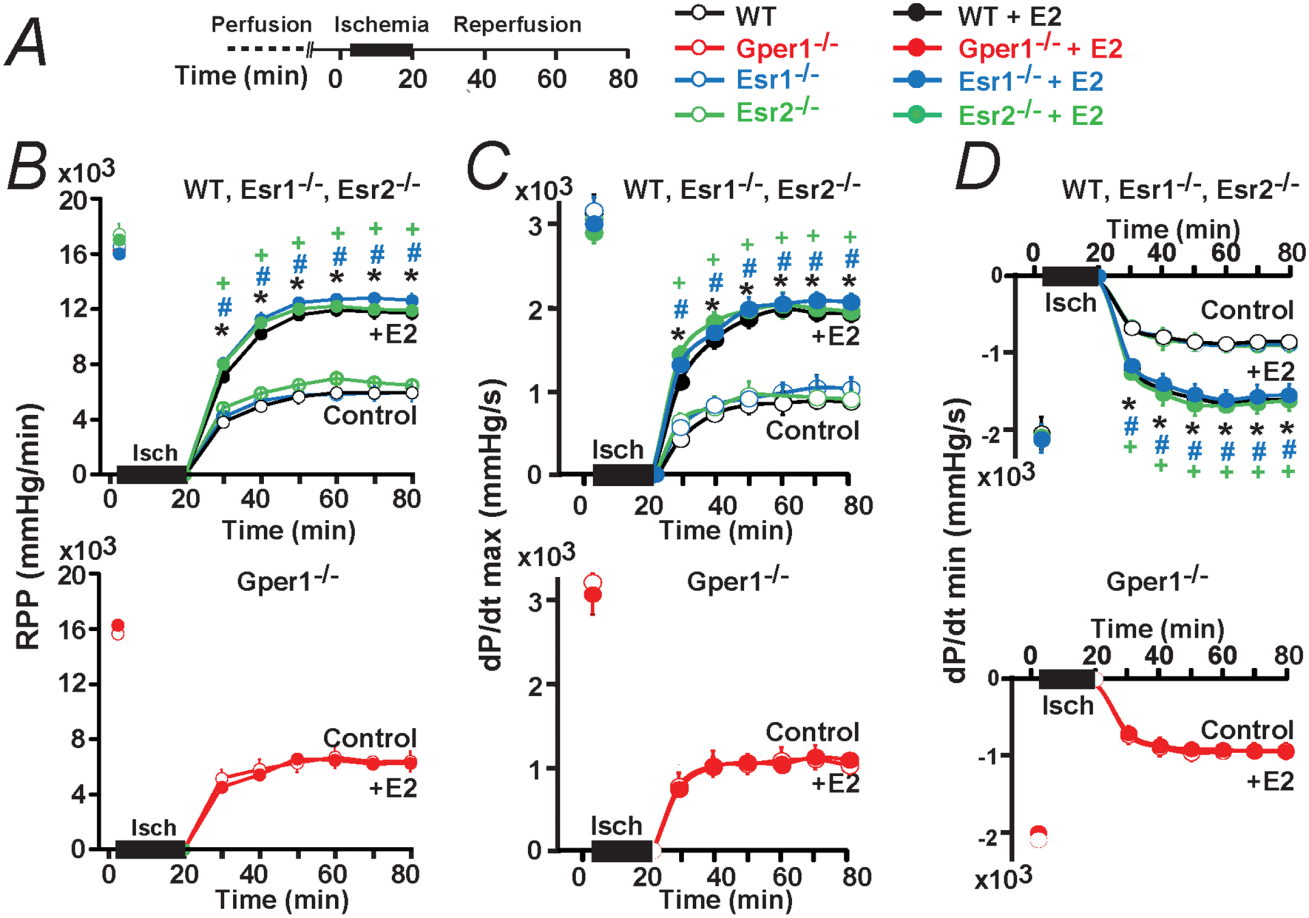
Time course of cardiac function after I/R in control and after E2 treatment in WT, Esr1^-/-^, Esr2^-/-^ and Gper1^-/-^. **A.** Ischemia/reperfusion (I/R) protocol used to perfuse mouse hearts isolated from WT, Esr1^-/-^, Esr2^-/-^ and Gper1^-/-^ male animals. **B-D.** Time course of Rate Pressure Product (RPP) (**B**), Maximum Velocity of Contraction (dP/dt max) (**C**) and Minimum Velocity of Relaxation (dP/dt min) (**D**). During the reperfusion period, E2 produced an improvement of all of the functional recovery parameters compared to control in WT, Esr1^-/-^, and Esr2^-/-^ (**B-D**, top); however, E2 had no effect when using Gper1^-/-^ hearts (B-D, bottom). Data points were obtained by averaging 2 min values before ischemia and every 10 min of reperfusion. Values are mean±SEM; * P<0.05 E2-treated *versus* control in WT; ^+^ P<0.05 E2-treated *versus* control in Esr2^-/-^, ^#^ P<0.05 E2-treated *versus* control in Esr1^-/-^. n = 6–8 hearts/ group.

Fig 2C and 2D illustrates that, when Esr1^-/-^ and Esr2^-/-^ hearts were used; acute E2 treatment had a remarkably similar protective effect as in WT animals. However, in hearts belonging to Gper1^-/-^ animals, E2 lost its ability to protect the heart against damages caused by I/R (Fig 2E). Mean values for rate pressure product (RPP), and velocity of contraction (dp/dt max) and relaxation (dp/dt min) during the reperfusion period are given in Fig 3B–3D confirming that WT hearts and hearts lacking Esr1 and Esr2 respond equally well to E2; while only Gper1 deficient hearts become impervious to the hormone. Other functional parameters are given in Table 1 as a function time.

**Table 1:** Heart recovery parameters by E2 in WT, Esr1^-/-^, Esr2^-/-^ and Gper1^-/-^.

Groups	LVSP (mmHg)	LVEDP(mmHg)	LVDP(mmHg)	HR(min)	Time
**WT**	97±5	2±0.5	95±5	172±3	Basal (before ischemia)
**WT+E2**	99±6	3±3	93±3	175±6	
**Esr1** ^**-/-**^	105±6	6±1	99±5	168±5	
**Esr1** ^**-/-**^ **+E2**	102±6	5±1	97±4	168±6	
**Esr2** ^**-/-**^	103±6	3±1	100±6	177±12	
**Esr2** ^**-/-**^ **+E2**	105±7	5±1	100±7	172±8	
**Gper1** ^**-/-**^	100±3	4±1	95±3	165±5	
**Gper1** ^**-/-**^ **+E2**	95±7	2±1	94±7	177±11	
**WT**	34±4	8±3	29±2	165±2	20 min (post-ischemia)
**WT+E2**	62±3 *	3±2	59±2 *	171±2	
**Esr1** ^**-/-**^	58±7	25±5	32±3	164±3	
**Esr1** ^**-/-**^ **+E2**	77±5 #	9±1 #	68±4 #	165±3	
**Esr2** ^**-/-**^	62±8	30±9	31±2	189±23	
**Esr2** ^**-/-**^ **+E2**	88±3 +	19±2	69±4 +	160±4	
**Gper1** ^**-/-**^	38±4	7±4	30±2	197±31	
**Gper1** ^**-/-**^ **+E2**	32±4	2±2	30±3	185±5	
**WT**	43±3	7±2	35±2	168±5	40 min(post-ischemia)
**WT+E2**	72±2 *	3±2	69±2 *	174±8	
**Esr1** ^**-/-**^	60±3	25±5	36±1	162±3	
**Esr1** ^**-/-**^ **+E2**	88±3 #	13±3	76±1 #	168±4	
**Esr2** ^**-/-**^	83±10	43±17	40±5	186±5	
**Esr2** ^**-/-**^ **+E2**	87±5	14±3	73±3 +	168±8	
**Gper1** ^**-/-**^	40±1	4±2	35±2	195±33	
**Gper1** ^**-/-**^ **+E2**	39±1	2±1	37±1	178±8	
**WT**	43±3	8±2	36±	167±5	60 min(post-ischemia)
**WT+E2**	72±5 *	3±2	69±2 *	171±5	
**Esr1** ^**-/-**^	63±6	26±6	37±1	163±8	
**Esr1** ^**-/-**^ **+E2**	83±2 #	11±2	75±1 #	169±5	
**Esr2** ^**-/-**^	79±12	39±15	39±4	166±5	
**Esr2** ^**-/-**^ **+E2**	86±2	14±4	72±3 +	164±4	
**Gper1** ^**-/-**^	36±2	4±1	32±1	175±18	
**Gper1** ^**-/-**^ **+E2**	37±2	2±2	35±2	173±5	

Cardiac functional parameters, left ventricular systolic pressure (LVSP); left ventricular end-diastolic pressure (LVEDP); left ventricular developed pressure (LVDP) and heart rate (HR) before ischemia (Basal) and at different times of reperfusion after ischemia with and without E2 treatment in WT, Esr1^-/-^, Esr2^-/-^ and Gper1. Values are mean±SEM.

* P<0.05 WT-control *versus* WT+E2-treated group (n = 4–6 hearts/ group)

^#^ P<0.05 Esr2^-/-^-control *versus* Esr1^-/-^+E2-treated group (n = 4–6 hearts/ group)

^+^ P<0.05 Esr2^-/-^-control *versus* Esr2^-/-^+E2-treated group (n = 4–6 hearts/ group)

**Fig 4:**
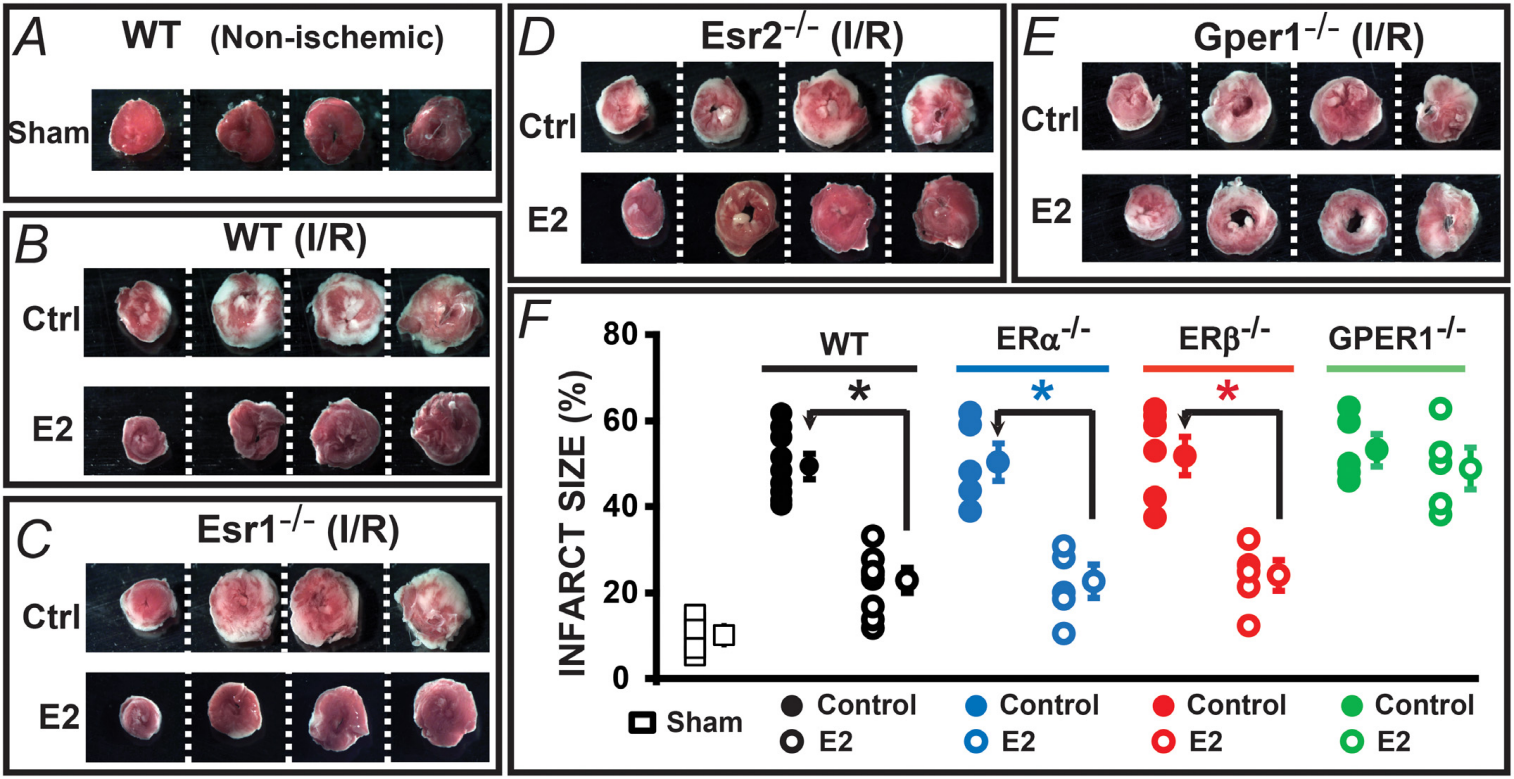
Gper1 activation is required to protect the heart from infarct induced by I/R. Hearts were treated as in Fig 2 and analyzed at the end of each protocol. **A-E.** Images are from slices of the same heart in each condition. White areas correspond to the infarcted zone. **A.** Infarct is minimal in isolated hearts not subjected to I/R. **B-D.** E2 protected equally well hearts from WT, Esr1 and Esr2 knockouts against infarct induced by I/R. **E.** Gper1^-/-^ was the only E2 receptor knockout examined where I/R caused the same degree of infarct in the presence or absence of E2. **F.** Individual and mean±SEM values of % infarct size. * P<0.05 control *versus* E2-treated groups (WT, n = 7–8 hearts/group; Esr1^-/-^, n = 5 hearts/group; Esr2^-/-^, n = 5–6 hearts/group). In Gper1^-/-^ hearts, E2 treatment did not reduce the infarct size (control = 52±3% *vs*. E2-treated = 47±4%, n = 6 hearts/group).

We also examined whether Gper1 expression is required for acute E2-induced improvement of heart viability by determining the myocardial infarct size in sham or hearts subjected to I/R as depicted in the protocols of Fig 3A. Hearts were sectioned and analyzed at the end of the perfusion (Fig 4A) or reperfusion periods (Fig 4B–4E). Fig 4A shows that perfused hearts not subjected to I/R had little damage over the 120 min perfusion period. In contrast, infarct size was substantial in hearts undergoing I/R in WT and knockout animals (Fig 4B–4E; control, ctrl). As expected, acute E2 treatment reduced myocardial infarct size significantly in WT animals (Fig 4B and 4F). Supporting the functional data, acute E2 treatment had the same protective action (as in the WT animals) when hearts from Esr1 and Esr2 knockouts were used (Fig 4C, 4D and 4F) but lost its protective effect in the absence of Gper1 as hearts from Gper1^-/-^ animals displayed a robust infarcted area regardless of E2 treatment (Fig 4E and 4F).

It is evident from the above results that Esr1 and Esr2 are dispensable for acute E2 protective action in male mice, whereas Gper1 expression is essential to sustain E2-mediated cardioprotection of hearts against I/R injury.

### Gper1 activation is required for E2-mediated increase in mitochondrial Ca^2+^ retention capacity

**Fig 5:**
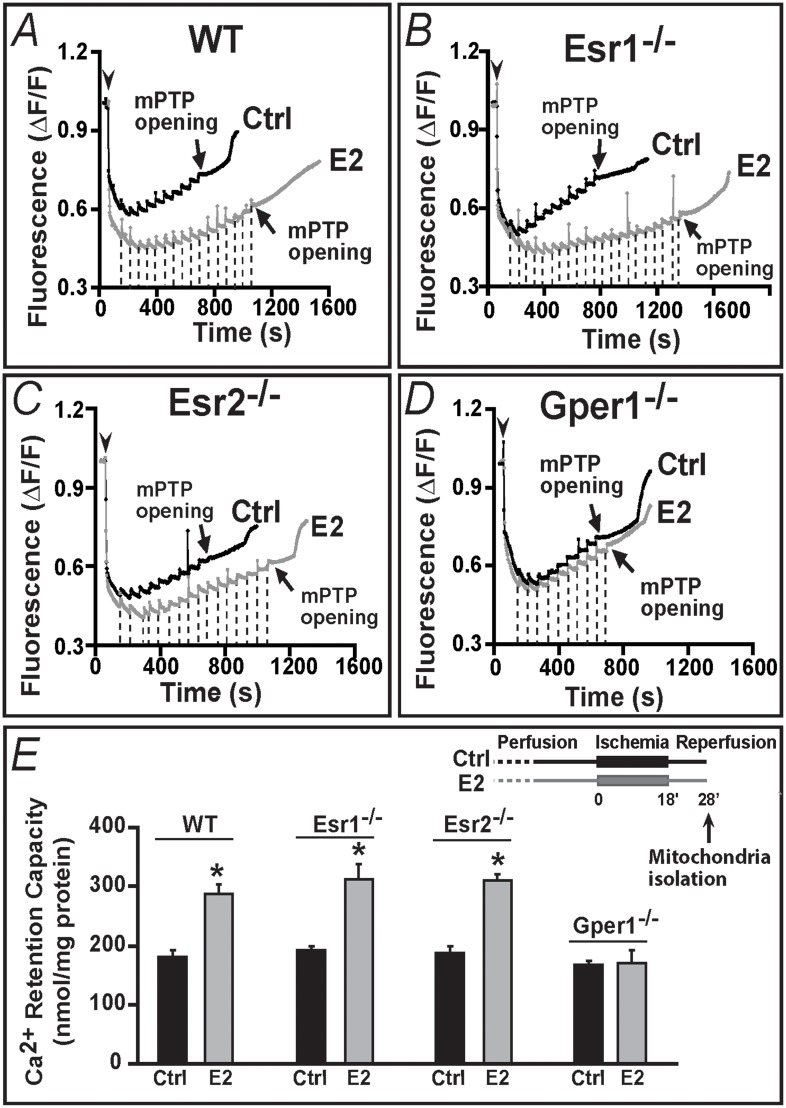
Gper1 activation is essential for E2 to induce increased resistance to Ca^2+^-overload of mitochondria from ischemic-reperfused hearts. **A-D.** Spectrofluorometric recordings of Ca^2+^ overload in mitochondria isolated from WT (**A**) Esr1^-/-^ (**B**), Esr2^-/-^ (**C**) and Gper1^-/-^ (**D**) hearts subjected to I/R (**E**, inset) in presence of vehicle (Ctrl, black traces) or E2 (gray traces). Arrowheads mark the time of mitochondria addition and the initial mitochondrial Ca^2+^ uptake. Subsequent 10 nmol Ca^2+^ pulses (dashed lines; only shown for E2 treated) were delivered until a spontaneous massive release was observed presumably to the opening of mPTP (arrows). Only mitochondria from Gper1^-/-^ lost their ability to endure higher Ca^2+^ overload by E2 treatment (**D**). **E.** Mean Ca^2+^ retention capacity values (amount of Ca^2+^ load needed to induce mPTP opening). Inset. I/R protocol marking the time of mitochondria isolation (10 min after reperfusion begun). Values are expressed as mean±SEM; * P<0.05 control *versus* E2-treated groups (n = 5 hearts/group).

We recently found that, a mechanism underlying the cardioprotective effect against I/R injury of Gper1 agonist, G1, is an increase in the Ca^2+^-load necessary to induce the mPTP opening [3]. Therefore, we reasoned that if indeed Gper1 is essential for E2 protective action, as concluded from the functional experiments in Figs 2–4, only mitochondria isolated from WT, Esr1^-/-^ and Esr2^-/-^ post-ischemic reperfused hearts should show an increased tolerance to Ca^2+^ overload or augmented Ca^2+^ retention capacity (CRC). To test this point, experiments were performed in mitochondria isolated at 10 min of post-ischemic reperfusion, a time when mPTP inhibition results in cardioprotection [24] (*see* Fig 5E, inset). Fig 5 shows that indeed only in WT, Esr1^-/-^, Esr2^-/-^ mitochondria-but not in Gper1^-/-^ mitochondria- acute E2 treatment increased mitochondrial Ca^2+^ load required to induce the mPTP opening, reflecting an inhibition of the mPTP opening. In all instances, mitochondria from control hearts required an average of nine to eleven 10 nmol Ca^2+^ pulses to trigger the mPTP opening (arrows) (Fig 5A–5D). This number was almost doubled when hearts were perfused with 40 nM E2 in WT, Esr1^-/-^, Esr2^-/-^ (Fig 5A–5C) but remained unchanged in mitochondria from Gper1^-/-^ animals (Fig 5D).

Mean values for mitochondrial CRC in each animal model in absence (control, ctrl) or presence of E2 are given in Fig 4E. All controls displayed similar values: WT = 180±12, Esr1^-/-^ = 193±7, Esr2^-/-^ = 187±10, and Gper1^-/-^ = 167±7 nmol/mg mitochondrial protein (n = 4 each). In contrast, E2 treatment produced a significant increase of this mitochondrial parameter only in WT, Esr1^-/-^ and Esr2^-/-^ to 287±17, 313±24; and 310±13 nmol/mg protein, respectively (n = 4 each), while it remained unchanged in Gper1^-/-^ (control, 167±7 vs. E2, 170±23 nmol/mg protein, n = 4 each). Together the results demonstrate the *sine qua non* nature of Gper1 in mediating E2-induced increase in mitochondrial CRC during reperfusion. The results also suggest that the Gper1-mediated cardioprotective effect of E2 is related to a decrease in mPTP sensitivity to Ca^2+^ overload.

### Signaling mechanisms involved in E2-mediated Gper1 activation: role of Akt, ERK1/2 and GSK-3β phosphorylation

**Fig 6:**
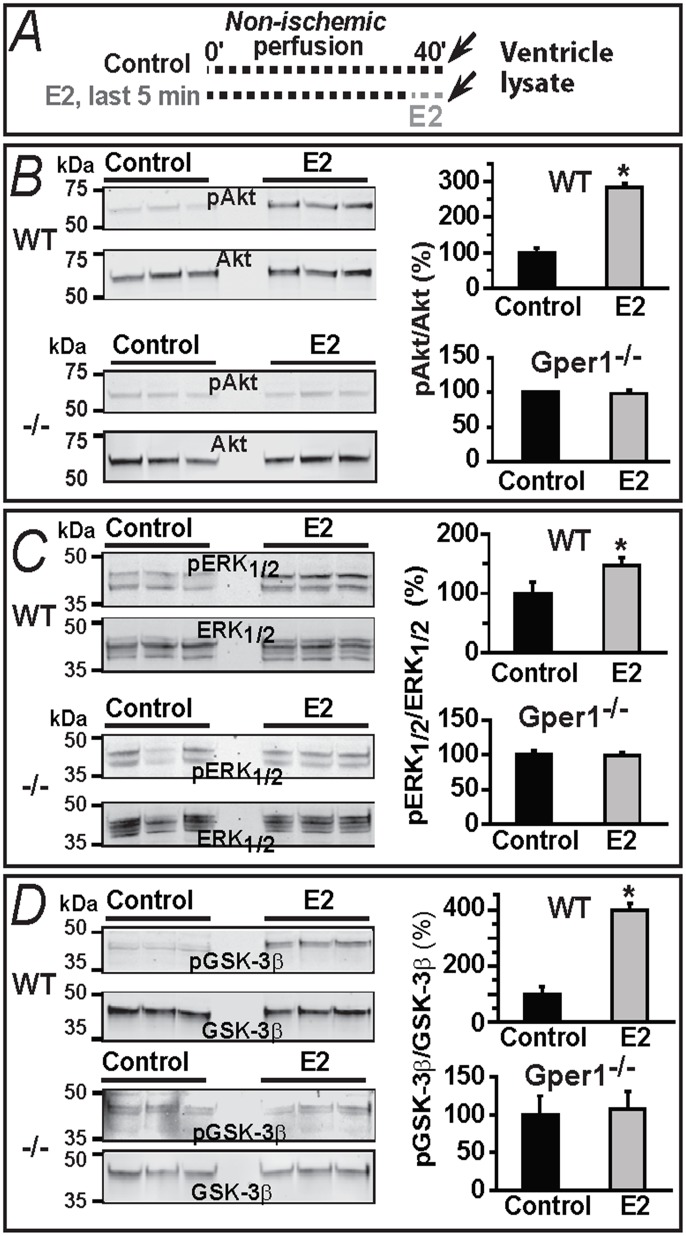
E2-induced increase in phosphorylation of Akt, ERK_1/2_ and GSK-3β is dependent on Gper1 gene expression and disappears with time for Akt and ERK_1/2_. **A.** Protocol used in panels **B,C,D**. Isolated hearts were either perfused 40 min with oxygenated KH solution or perfused with oxygenated KH for 35 min and for 5 min with KH plus 40 nM E2. **B-D.** Immunoblots and corresponding bar graphs show that 5 min “pre-ischemic” treatment with E2 provoked pAkt/Akt, pERK_1/2_/ERK_1/2_ and pGSK-3β/GSK-3β ratios to increase in WT but not in Gper1^-/-^ samples. Control ratios were set to 100%. Values are mean±S.E.M.; *, P<0.05 control *versus* E2-treated group, n = 7 hearts/group.

Previous work has shown that in a *non-ischemic* Langedorff preparation at least two signaling kinases-Akt and ERK- get activated by stimulating Gper1 with the agonist, G1, for 10 min [9]. However, phosphorylation of these kinases or that of GSK-3β -an end-point integrator kinase involved in mPTP inhibition [25]- has not been investigated for the acute E2-Gper1-selective activation. To address this point, we compared in WT and Gper1^-/-^ hearts the phosphorylation levels of the three enzymes using the protocols depicted in Fig 6A. Confirming the essential role of Gper1 in acute E2 stimulation, phosphorylated (p) Akt, pERK1/2 and pGSK-3β immunoblot signals (expressed as a % of the total levels of each enzyme) were increased by 5 min treatment with 40 nM E2 in the *pre-ischemic* hearts (Fig 6B–6D) of WT but not of Gper1^-/-^ mice. These results suggest that acute E2-Gper1-induced cardioprotection action results in an activation of Akt and ERK_1/2_ and deactivation of GSK-3β.

### E2 triggers ERK_1/2_ and Akt parallel pathways but only ERK_1/2_ pathway converges on GSK-3β

**Fig 7:**
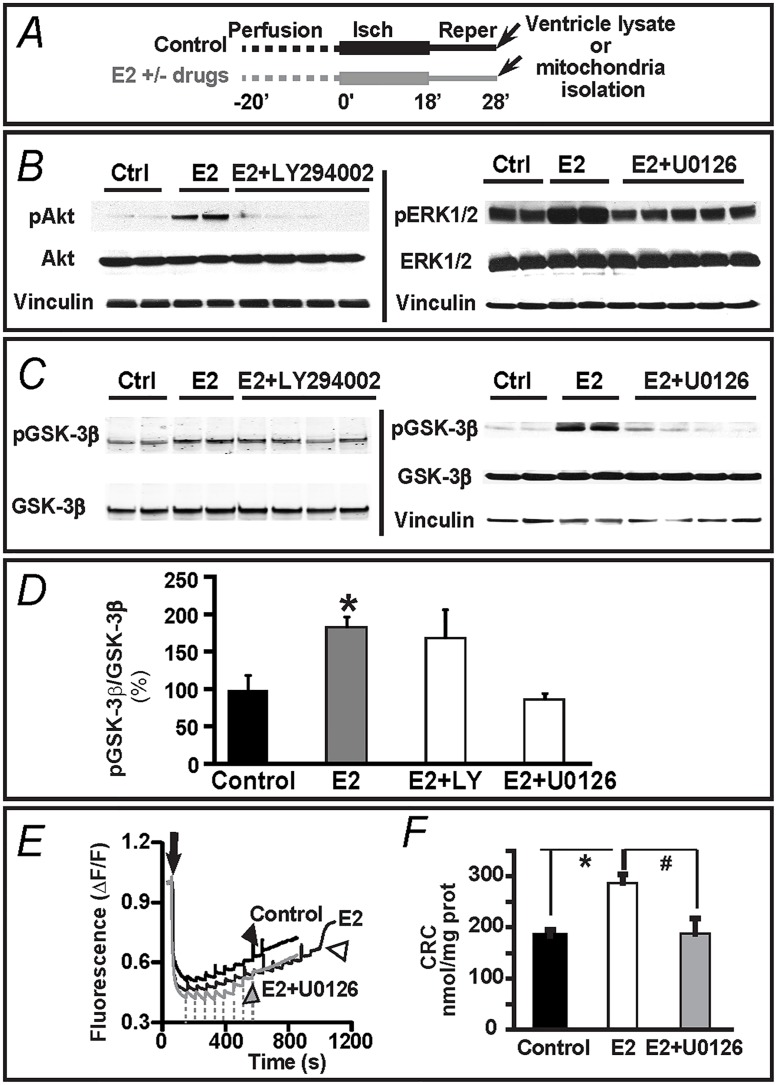
MEK_1/2_/ERK_1/2_ –but not PI-3K/Akt- signaling supports E2-mediated protection from I/R via GSK-3β and mPTP. **A.** I/R protocol. Arrows mark the time of sample preparation. **B.** Immunoblots show that addition of the inhibitor of MEK_1/2_/ERK_1,2_ pathway, U0126 (1 μM) abolishes E2-induced up-regulation of pERK and the inhibitor of PI3-K/Akt pathway, LY294002 (10 μM) abolishes the increase in pAkt induced by 40 nM E2. **C,D.** Immunoblots and corresponding bar graphs show that the inhibitor of MEK_1/2_/ERK_1,2_ pathway, U0126 (1 μM)-but not the inhibitor of PI3-K/Akt pathway, LY294002 (10 μM)- abolishes the increase in pGSK-3β/GSK-3β ratio induced by 40 nM E2. **E.** Calcium load measurements were as indicated in Fig 5. Arrow, addition of mitochondria. Arrowheads, mark the massive Ca^2+^ release (an index of mPTP opening) in mitochondria from control (black), E2 (40 nM)-treated (open), and treated with E2 (40 nM) + U0126 (1 μM) (grey) hearts. Dashed lines mark the time of Ca^2+^ addition to the E2+U0126 treated sample. **F.** Mean values of calcium retention capacity (CRC) demonstrate that 1 μM U0126 prevents the beneficial effect of 40 nM E2. Note that vinculin was used as a loading housekeeping protein. All values were obtained from WT male mice and expressed as mean±SEM; * P<0.05 E2-treated group *versus* control; and # P<0.05 E2+U0126 *versus* E2-treated group (n = 5–7 hearts/group).

We next investigated, whether the activation of Akt and/or ERK_1/2_ signaling by acute E2-Gper1 action found in Fig 6B and 6C lead to GSK-3β deactivation. Having demonstrated that Gper1 activation a key role for acute E2-mediated cardioprotective effects in male mice, the rest of the experiments were performed using male WT animals. The levels of GSK-3β phosphorylation were measured in ventricle lysates at the end of the 10 min reperfusion period, as indicated in Fig 7A, from hearts that were perfused with vehicle (control) or E2 in the absence or presence of selective inhibitors of the PI-3K/Akt (LY294002, 10 μM) and MEK_1/2_/ERK_1/2_ (U0126, 1 μM) pathways [26,27]. Fig 7B show that addition of the inhibitor of MEK_1/2_/ERK_1,2_ pathway, U0126 (1 μM) abolishes E2-induced up-regulation of pERK and the inhibitor of PI3-K/Akt pathway, LY294002 (10 μM) abolishes the increase in pAkt induced by 40 nM E2. Fig 7C and 7D shows that the increase in GSK-3β phosphorylation (normalized to total GSK-3β) induced by E2 was practically unaffected by inhibiting the PI-3K/Akt pathway with LY294002 (n = 4). In contrast, inhibiting MEK_1/2_-dependent phosphorylation of ERK_1/2_ with U0126 produced a total ablation of GSK-3β phosphorylation to control levels (n = 6). In conjunction with the results in Fig 6, these data reveal that E2-mediated Gper1 activation triggers at least two early (within 5 min) and parallel pathways, one involving MEK_1/2_-ERK_1/2_ phosphorylation upstream of GSK-3β, and another related to PI-3K/Akt signaling that is independent of GSK-3β phosphorylation.

Phosphorylated GSK-3β is known to inhibit the mPTP in response to a variety of cardioprotective agents [25]; thus, we tested whether this mechanism also applies to the acute E2-Gper1 mediated cardioprotection through MEK_1/2_-ERK_1/2_–GSK-3β pathway. To this end, we evaluated mPTP inhibition using as parameter the CRC of mitochondria from pre-ischemic reperfused hearts, as in Fig 5. Hearts were treated either with vehicle (control), 40 nM E2, or with 40 nM E2 plus 1 μM of the MEK_1/2_ inhibitor, U0126; mitochondria were isolated at 10 min of reperfusion (Fig 7A). Calcium loading measurements in Fig 7E demonstrated that in the presence of U0126 (gray trace), the number of Ca^2+^ pulses (dashed lines) needed to open the mPTP (gray arrowhead) resembled the non-protected condition (control; black arrowhead); thus the drug was able to abolish the E2-mediated increased tolerance to Ca^2+^ overload. The mean values for the three conditions (Fig 7F; n = 5), clearly demonstrate that inhibiting MEK_1/2_-ERK_1/2_ signaling with U0126 prevents the E2-enhanced mitochondrial CRC or E2-mediated inhibition of mPTP opening. Taken together, the results in Figs 5–7 support the view that the activation of the MEK_1/2_-ERK_1/2_ pathway-triggered by acute E2-activated-Gper1- is capable to control the phosphorylation of GSK-3β and as consequence the activity of the mitochondria permeability transition pore. Whether this pathway or the alternative E2-Gper1-Akt pathway is a key to E2-mediated cardioprotection was examined next.

### Which of the E2-Gper1 activated kinase pathways, ERK_1/2_ and/or Akt, is key to prevent heart injury?

**Fig 8:**
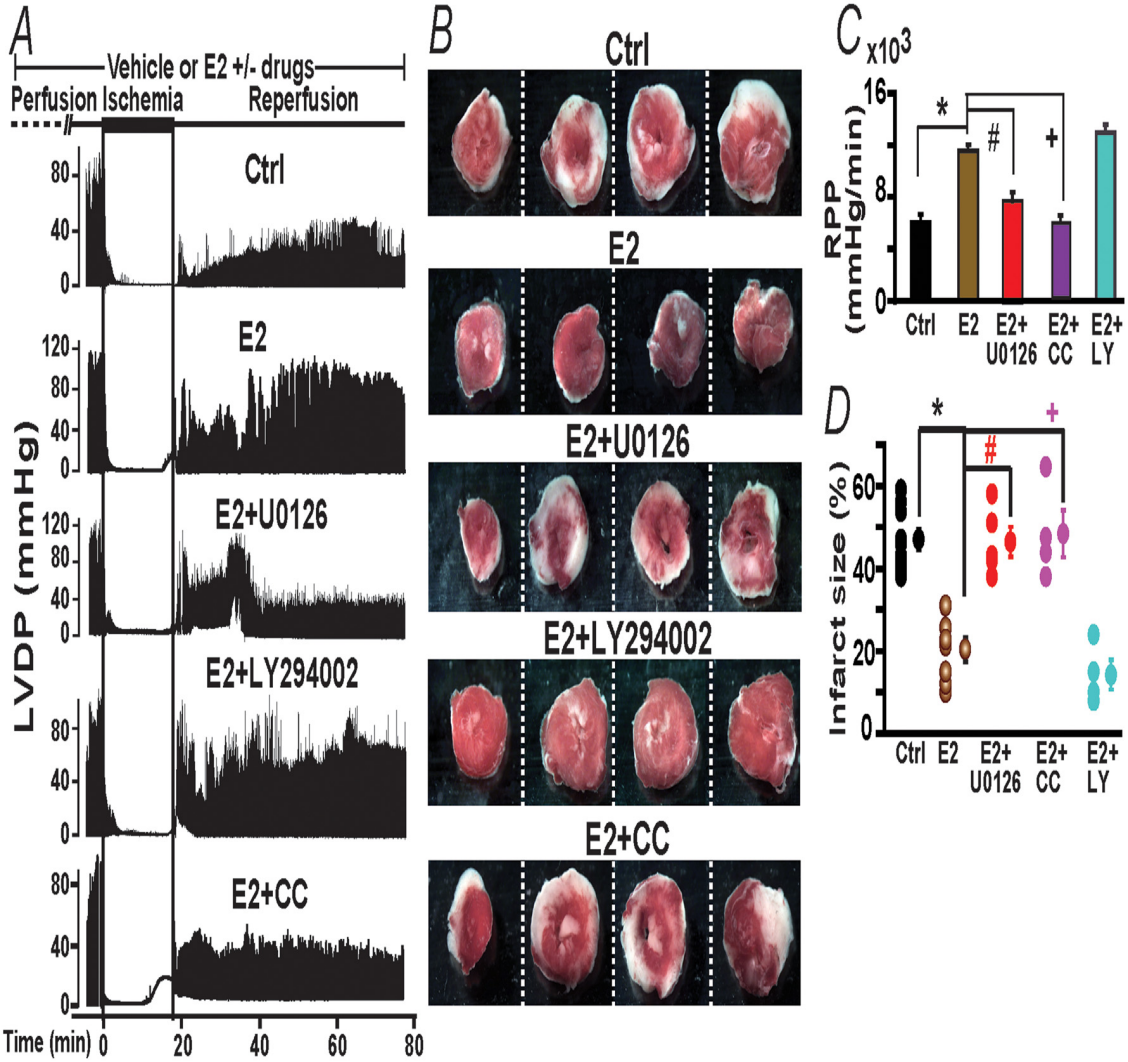
E2-induced cardiac protection against I/R injury is prevented by inhibitors of PKC translocation and MEK_1/2_ but not by a PI-3K inhibitor. **A.** Time course of LVDP changes by E2 +/- drugs during the I/R protocol (scheme at top). E2-induced protection during reperfusion was diminished by cotreatment during the whole protocol with MEK_1/2_ inhibitor, U0126 and with PKC translocation inhibitor, chelerythrine chloride (CC) but not with PI-3K inhibitor, LY294002. **B.** Heart sections of same heart in each condition stained at the end of the reperfusion period. White areas are infarcted zones. LY294002 co-treatment failed to inhibit E2-mediated prevention of heart infarct. **C,D,E.** Mean values of Rate Pressure Product (RPP), and % infarct size in control hearts (ctrl, perfused with vehicle) and hearts treated with E2 (40 nM), E2 (40 nM)+U0126 (1 μM), E2 (40 nM)+LY294002 (10 μM), and E2 (40 nM)+CC (1 μM). RPP and dP/dt max were obtained by averaging the last 2 min of reperfusion. Values are expressed as mean±SEM; *, P<0.05 E2-treated group *versus* control (n = 6-8/group); #, P<0.05 E2+U0126 *versus* E2-treated group; +, P<0.05 E2+CC *versus* E2-treated group (n = 4-7/group). Functional values as a function of time are given in Table 2. All values were obtained using WT male mice.

To address this question, we directly tested whether inhibiting MEK_1/2_-ERK_1/2_ or PI-3K/Akt pathways can suppress acute E2-induced functional improvement and protection against myocardial infarct. Fig 8A shows typical LVDP traces as a function of time in control (vehicle treated), and E2 treated hearts in the absence or presence of kinase inhibitors; while Fig 8B shows the degree of myocardial infarction produced by each treatment at the end of the reperfusion period. Both parameters were affected in similar ways by the pharmacological maneuvers. Inhibition of MEK_1/2_-ERK_1/2_ by continuous perfusion of 1 μM U0126 (+E2) was able to effectively inhibit the E2-mediated increase in heart activity during reperfusion, as well as to inhibit the reduction in infarct size by E2 with respect to controls. In marked contrast, inhibition of the PI-3K/Akt pathway with 10 μM LY294002 could not prevent E2-mediated protection neither in function nor in the degree of myocardial infarct.

To further investigate the MEK_1/2_-ERK_1/2_ pathway, we also inhibited protein kinase C (PKC) translocation an upstream event in cardiomyocyte protection by activated adenosine A1 and A2b receptors [25,28,29]. The bottom panel in Fig 8A and 8B clearly show that inhibiting PKC translocation with 1 μM chelerythrine chloride (CC) [30] completely reversed the beneficial effect of E2, causing a significant decrease in LVDP and myocardial infarct size during reperfusion similar to the levels attained in the control, without E2. Likewise, the presence of the PKC inhibitor caused a marked increase in infarct size resembling that attained in the absence of E2 (control). Mean values of functional parameters, RPP as well as for myocardial infarct size in each condition are given in panels C-D; other functional parameters are given in Table 2.

**Table 2:** Heart functional recovery parameters by E2 and in presence of MEK_1/2_, PKC translocation and PI-3K inhibitors in WT mice.

Groups	LVSP (mmHg)	LVEDP(mmHg)	LVDP(mmHg)	HR(min)	Time
**Control**	97±5	4±1	93±5	176±9	Basal
**E2**	100±6	2±2	97±3	169±8	
**E2+U0126**	106±5	7±3	99±2	166±15	
**E2+LY294002**	109±12	8±1	101±12	153±12	
**E2+CC**	92±12	2±1	90±13	210±21	
**Control**	37±3	9±2	28±2	167±17	20 min
**E2**	65±4 *	2.5±2	63±4 *	160±8	
**E2+U0126**	57±9	16±5	39±9 #	194±21	
**E2+LY294002**	67±4	8±1	59±4	139±19	
**E2+CC**	37±7 +	12±8	25±3 +	220±37	
**Control**	45±3	9±2	36±2	167±15	40 min
**E2**	71±2 *	3±1	68±2 *	173±6	
**E2+U0126**	66±14	23±17	43±7 #	200±22	
**E2+LY294002**	81±17	8±1	73±16	175±39	
**E2+CC**	45±21 +	13±9	33±5 +	211±29	
**Control**	43±2	9±2	34±2	179±14	60 min
**E2**	71±5 *	3±2	68±2 *	168±4	
**E2+U0126**	72±11	22±16	49±6 #	180±34	
**E2+LY294002**	81±10	10±3	71±8	188±16	
**E2+CC**	50±12 +	13±1	37±5 +	160±9	

**Cardiac functional parameters in WT male mice.** Left ventricular systolic pressure (LVSP); left ventricular end-diastolic pressure (LVEDP); left ventricular developed pressure (LVDP) and heart rate (HR) before ischemia (Basal) and at different times of reperfusion after I/R in control, E2-treated, and E2+Inhibitors (U0126, LY294002 and CC: chelerythrine chloride). Values are mean±SEM.

* P<0.05 control *versus* E2 group (n = 6-7/ group)

^+^ P<0.05 E2+CC *versus* E2 group (n = 4-6/ group)

^#^ P<0.05 E2+U0126 *versus* E2 group (n = 4–6 hearts/ group)

Taken together, the results discarded a primordial beneficial effect of the activated PI-3K/Akt pathway in mediating the acute E2-induced cardioprotection after I/R in male mice. Rather, they are consistent with the idea that the main salvatory pathway triggered by acute E2-Gper1 stimulation is the activation of MEK_1/2_-ERK_1/2_ (putatively linked to PKC translocation) signaling that leads to GSK3-β phosphorylation and ultimate target, the inhibition of the mPTP opening.

## Discussion

In this study, we discovered that in male mice: 1) Gper1 activation is essential for the cardioprotective action of acute E2 on the perfused heart subjected to I/R, and that Esr1 and Esr2 are dispensable to this effect; and 2) although both PI-3K/Akt and MEK_1/2_/ERK_1/2_ pathways are initially (prior ischemia) triggered by E2-Gper1 activation, only the MEK_1/2_/ERK_1/2_ pathway transcends in mediating I/R cardioprotection via GSK3-β phosphorylation and regulation of the mPTP opening.

### Gper1 vs. Esr1 and Esr2 role in mediating the ‘acute’ E2 protective action in I/R

Pharmacological stimulation of the three ER receptors, Gper1, Esr1 and Esr2 have indicated that the three receptors play a role in cardioprotection against I/R injury in the rat and mouse models. Specifically, stimulation during reperfusion with agonists of Esr1 and Esr2 (PPT and DPN, respectively) result in heart protection of male rats using the same Langendorff model [6] as in studies in male mice and rats of both genders testing Gper1 with G1 [3,31]. However, using female rabbits and a different model of I/R (in-vivo) it was shown that Esr2 stimulation with the same drug caused no protective action [7]. Obviously, differences in species and protocols used, or unknown actions of the drugs could be compounding factors causing these different results.

In this work, the systematic analysis of WT, Esr1, Esr2 and Gper1 knockout mice led to the discovery that Gper1 expression is essential for the acute action of E2 to protect the isolated male heart from I/R injury. Only the Gper1^-/-^ -but not Esr1^-/-^ nor Esr2^-/-^- mice showed complete lack of E2 beneficial effect, including the increased mitochondria tolerance to Ca^2+^ overload in the ischemic reperfused heart (Figs 2–4). Although in the heart of WT male mouse the three receptors likely coexist (Fig 1) and could be activated by E2, our results strongly support the idea that Gper1 activation plays a preponderant role in protecting the male heart against I/R damage. A key role of Gper1 over Esr1 has also been proposed in the liver where suppression of Gper1 but not of Esr1 expression with siRNA prevented E2-induced beneficial action after trauma-hemorrhage injury [32].

### E2-Gper1 signaling mechanisms in the heart of male mouse

Until now, Gper1 signaling mechanisms in the heart have been seldom addressed and with apparent discrepancies as to the role of ERK in mediating protection from I/R by the Gper1 agonist, G1 [3,9]. As recent studies have shown that G1 may have alternative effects independent of Gper1 [33], it was indispensable to clarify the involvement of this pathway in Gper1-associated signaling.

We now provide new signaling information to understand how the acute activation of Gper1 by E2 protects the male mouse heart from ischemic insult. Using the Gper1^-/-^ mouse, our studies revealed that pre-ischemic stimulating Gper1 with E2 for 5 min triggers the phosphorylation of salvage kinases Akt, and ERK_1/2_. Notably, when inhibiting ERK_1/2_ phosphorylation by MEK_1/2_ using U0126 during the entire protocol, we found that E2 was no longer capable of producing protection (Fig 7) indicating that MEK_1/2_/ERK_1/2_ pathway activation is involved in acute E2- Gper1 action. This result confirms our previous data showing that PD-98059, a MEK_1/2_-ERK_1/2_ pathway inhibitor, is able to prevent G1-induced protection when applied during the whole I/R protocol [3] as in the present studies. Our results differ from those obtained in the rat heart where PD-98059 was unable to prevent G1-induced protection measuring the same parameters, although ERK_1/2_ phosphorylation augmented [9]. This disparity could be explained by a species difference (rat vs. mouse) or by the usage of a distinct protocol as in those studies drugs were applied for only 10 min prior ischemia, whereas in our studies they were applied for the duration of the protocol. Whether acute E2 treatment throughout the experiment, like used in our protocol, causes that ERK_1/2_ phosphorylation to turn on explains why U0126 prevents E2-induced protection, is still an open question. Nonetheless, it is reasonable to conclude that MEK_1/2_-ERK1/2 pathway is indeed involved in the protection of the male mouse heart from I/R injury by continuous stimulation of Gper1 by acute E2 treatment.

On the other hand, the activation of Akt was not sufficient to explain acute E2-Gper1-induced protection because co-administration of the hormone and LY294002 (a PI-3K/Akt pathway inhibitor) during the whole protocol could not prevent the decrease in functional recovery or in infarct size induced by acute E2 treatment. The absence of the effect of LY294002 at 10 mM on acute E2 cardioprotection cannot be related by its dose used as we found that addition of LY294002 prevented E2-induced up-regulation of phosphorylation of Akt (pAkt) observed in WT perfused heart (Fig 7B). Our observation is in contrast to the conclusion reached using 100 nM Wortmannin to probe the role of PI-3K/Akt pathway in the rat I/R model pre-conditioned with G1 [31]. In these studies, possible explanations to the apparent discrepancy are species-related differences or that inhibition of G1-induced protection by 100 nM Wortmannin results from inhibition of enzymes other than PI-3K like phospholipase C (PLC) (Wortmannin IC_50_ for PLC = 28 nM) [34], which would prevent activation of the PLC/PKC pathway indicating that wortmannin is not specific to PI-3K [34]. We favor the latter explanation as in our experiments acute E2-induced protection could also be prevented by the inhibitor of PKC translocation, CC (Fig 8). Our results also demonstrate that the participation of Akt in the acute E2-induced cardioptotection, as reported here, is different to that observed for long term effects of E2, where genomic effects likely occur. For example, treatment of E2 for 7 days induced an increase of pAkt measured at 6 hr post-infarction in an *in vivo* model [35]; and E2 deficiency in ovariectomized mice results in reduced levels of pAkt at the end of the I/R injury protocol [36]. At present, the precise role of the Gper1-dependent initial activation of Akt by acute E2 treatment remains an open question (Fig 8); however, we can conclude that in contrast to the MEK_1/2_-ERK_1/2_ pathway, the PI-3K/Akt pathway is not critical in mediating the cardioprotective effects that acute E2-Gper1 activation has on I/R injury.

Consistent with the above conclusion, the MEK_1/2_-ERK_1/2_ (but not the PI-3K/Akt) salvatory pathway triggered by E2-Gper1 activation was found to be upstream of GSK-3β and having as final effect the reduction in mPTP activity (Figs 6, 7 and 8). Our results are consistent with previous results showing that several cardioprotective agents/maneuvers have as common downstream target GSK-3β, whose phosphorylation and deactivation results in the inhibition of mPTP opening [25,37], even though the requirement of GSK-3β inactivation in preconditioning and postconditioning mechanism has been challenged [38]. Our findings that inhibition of PKC translocation abolished acute E2 beneficial effect on heart functional recovery (Fig 8) and that Gper1 is the main acute E2 target protecting the heart from I/R insult (Figs 3, 4 and 5) lead us to propose that E2-Gper1 protective pathway in the mouse male heart involves PKC. Supporting this view, PKC has been recently implicated in the G1-mediated stimulation of Gper1 in the perfused kidney [39].

**Fig 9:**
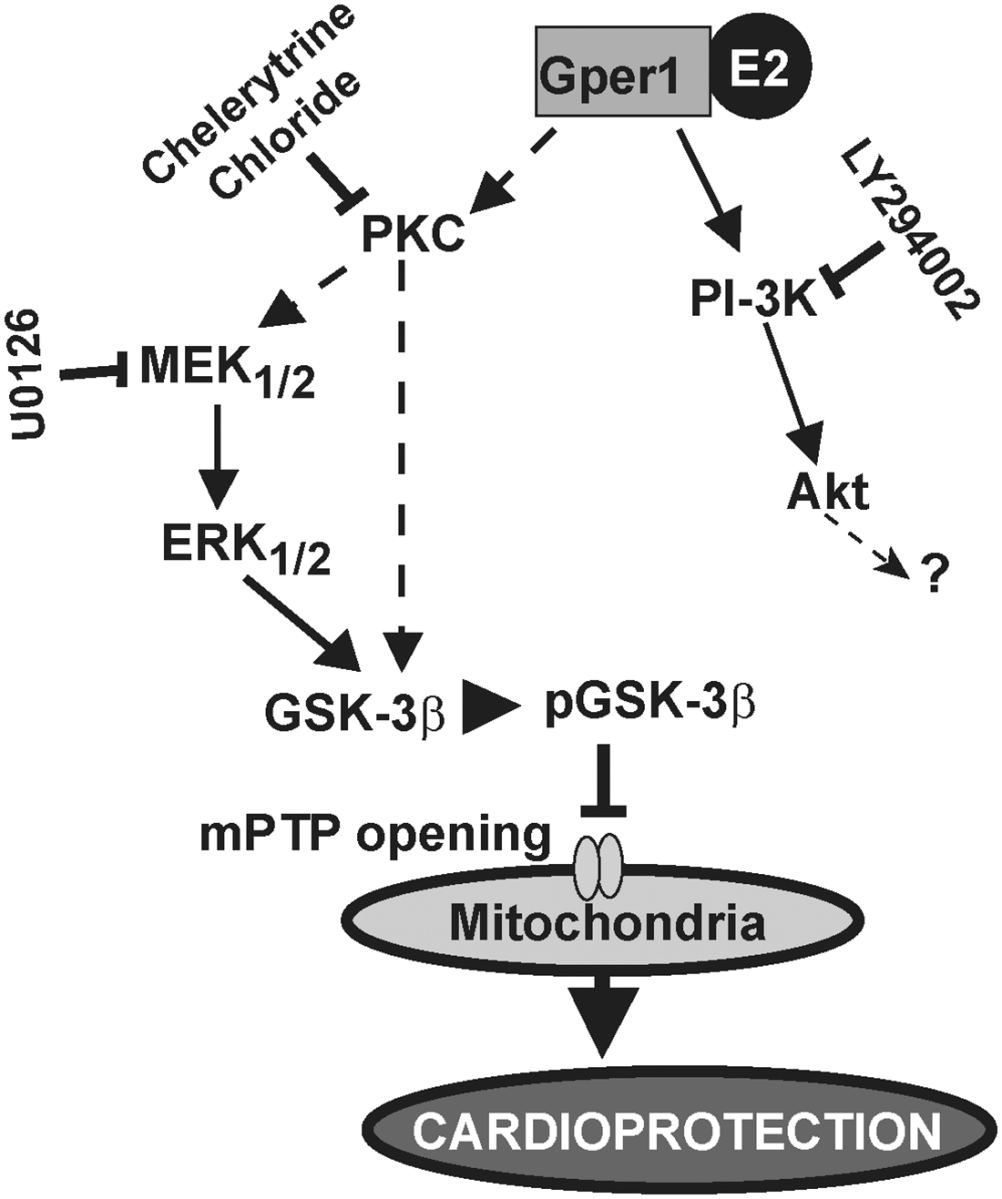
Salvage mechanisms triggered by E2 via Gper1 during I/R. E2 binding to Gper1 may initially trigger protein kinase C (PKC) translocation. PKC could directly or via activation of MEK_1/2_/ERK_1/2_ pathway increase phosphorylation of GSK-3β, which in turn would inhibit mPTP opening resulting in cardioprotection. Chelerythrine chloride, inhibitor of PKC translocation; U0126, inhibitor of the MEK_1/2_/ERK_1/2_-pathway; and LY294002, inhibitor of PI-3K. E2 through Gper1 can also induce a transient activation of PI-3K/Akt pathway, but this activation does not play an important role in the acute E2 induced cardioprotection after I/R. Black arrows, pathways demonstrated in this work. Dashed arrows, putative pathways.?, unknown target.

In resemblance to the pathways triggered by adenosine A1 receptor in cardiomyocytes [25], PKC could activate the MEK_1/2_-ERK_1/2_ cascade (which in turn and according to our studies induces GSK3-β phosphorylation preventing opening of mPTP), or act directly on GSK3-β to prevent mPTP opening (Fig 9). The protective action of Gper1 differs from that of A1 and bradykinin receptors [40,41] in that E2-Gper1 beneficial effect on heart function during I/R involving phosphorylation of GSK3-β does not depend on Akt activation (Figs 8–9).

In the present study, we have used chelerythrine chloride, 1,2-Dimethoxy-12-methyl [1,3] benzodioxolo[5,6-*c*]phenanthridinium chloride, as an inhibition of PKC. PKC enzymes are involved in controlling the function of other proteins through the phosphorylation of hydroxyl groups of serine and threonine amino acid residues on these proteins. Therefore, PKC enzymes play important role in several signal traduction cascades. Although our results clearly show the involvement of PKC in Gper1 action, further experiments are needed to determine which PKC isoform (s) is/are involved in Gper1 mechanism. Given that Gper1 activation results in mitochondrial calcium regulation, it would be interesting to identify among the classical PKC isoforms α, β_I_, β_II_, and γ that require Ca^2+^ for activation and novel isoforms δ, ε, η, and θ.

In conclusion, our studies provided the first evidence for a key role of Gper1 (but not Esr1 nor Esr1) in mediating the acute E2 protection against I/R injury in male mice heart, and highlighted particular features of acute E2-Gper1 signaling pathway in the heart. Specifically, results revealed Gper1 coupling to PKC in the heart, likely signaling through the MEK_1/2_-ERK_1/2_-GSK-3β pathway to inhibit the mPTP and cause cardiac protection. Further, we demonstrated Akt activation as dispensable for acute E2 protection against I/R reperfusion injury in the male heart. Finally, our findings raise the intriguing possibility to therapeutically target Gper1 in a clinical setting in order to protect against acute myocardial infarction.

The conclusions reached in this study apply only to the male mouse heart. Further investigations in female mouse hearts need to be performed for comparison. We are aware that pharmacologic agents used to inhibit the different pathways may have off target effects at certain doses; therefore, we have used only concentration already applied in our previous studies to minimize their degree on non-specific targets. In the present studies, we measured infarct size using TTC staining, which in small heart may be not accurate; we will consider using enzymatic methods, such as LDH or CK measurements of the effluent, in our further study to support the present observations.
